# A boronic acid-functionalized phthalocyanine with an aggregation-enhanced photodynamic effect for combating antibiotic-resistant bacteria†
†Electronic supplementary information (ESI) available. See DOI: 10.1039/d0sc01351j


**DOI:** 10.1039/d0sc01351j

**Published:** 2020-05-18

**Authors:** Eunhye Lee, Xingshu Li, Juwon Oh, Nahyun Kwon, Gyoungmi Kim, Dongho Kim, Juyoung Yoon

**Affiliations:** a College of Chemistry , State Key Laboratory of Photocatalysis on Energy and Environment , Fujian Provincial Key Laboratory of Cancer Metastasis Chemoprevention and Chemotherapy , Fuzhou University , Fuzhou 350108 , China . Email: xingshuli@fzu.edu.cn; b Department of Chemistry and Nanoscience , Ewha Womans University , Seoul 120-750 , Republic of Korea . Email: jyoon@ewha.ac.kr; c Spectroscopy Laboratory for Functional π-Electronic Systems , Department of Chemistry , Yonsei University , Seoul 120-749 , Republic of Korea . Email: dongho@yonsei.ac.kr

## Abstract

Most existing photosensitizers (*e.g.*, porphyrins) are often aggregated in aqueous solution because of their large conjugated molecular structures. This aggregation usually results in a lack or low levels of reactive oxygen species (ROS) generation due to aggregation-caused quenching, which severely hampers the application of photosensitizers in photodynamic therapy (PDT). Herein, we make an interesting finding that a boronic acid-functionalized phthalocyanine (PcN4-BA) displays an uncommon phenomenon, an aggregation-enhanced photodynamic effect. The combination of the ability to form uniform nanostructured self-assemblies in water, highly efficient ROS generation and boronic acid-induced targeting give PcN4-BA excellent performances in antimicrobial PDT.

## Abstract 2

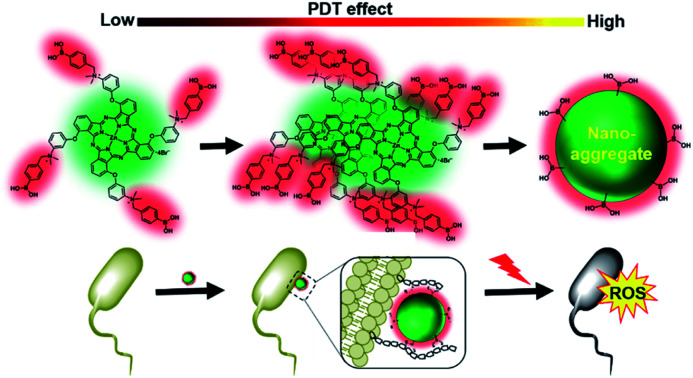
A boronic acid functionalized phthalocyanine displays aggregation-enhanced reactive oxygen species (ROS) generation and excellent photodynamic antibacterial activity.

## Introduction

Bacterial infection has posed a grave challenge to the lives and health of human beings. In particular, the growing number of antibiotic-resistant bacteria have complicated infection treatments, and some infections (*e.g.*, those caused by superbugs) now fail to respond to traditional first-line treatments.^1^–^4^ This promotes a search for new therapeutic strategies. Photodynamic therapy (PDT) is an effective and alternative method for infection treatment. PDT utilizes a photosensitizer (PS) under light exposure to initiate a cascade of photochemical reactions generating reactive oxygen species (ROS) responsible for bacterial inactivation.^5^–^8^ A combination of spatiotemporal selectivity, an enhanced immune response and the ability to perform repetitive treatments without resistance endow PDT with great antimicrobial potential.^9^–^11^ The full promise of antimicrobial PDT, however, has not yet been realized. This is mainly associated with the lack of ideal PSs and efficient delivery processes. Most PSs (*e.g.*, porphyrins) are large conjugated compounds that often strongly aggregate in aqueous solution, leading to insufficient ROS generation because the aggregation causes quenching. Another problem concerns the low targeting ability that current PSs have for bacterial cells.

**Fig. 1:**
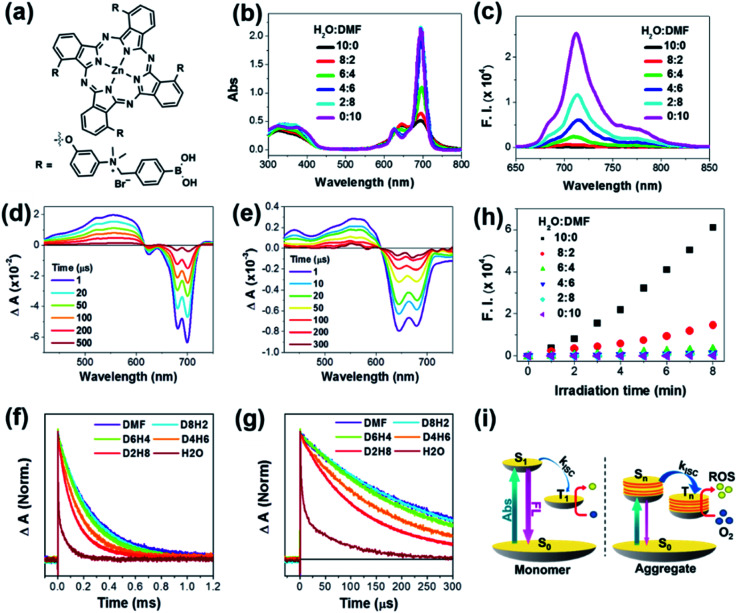
Aggregation-enhanced photodynamic effect of PcN4-BA. (a) Molecular structure of PcN4-BA. (b) Absorption and (c) emission (excited at 635 nm) spectra of PcN4-BA (10 μM) in a mixed solution of DMF and H_2_O with various ratios. TA spectra of PcN4-BA in (d) DMF and (e) H_2_O and (f and g) normalized decay profiles in mixed solutions of DMF and H_2_O at various ratios (excited at 680 nm). D8H2 indicates that the ratio of DMF : H_2_O is 8 : 2. (h) ROS generation plot of PcN4-BA in mixed solutions of DMF and H_2_O at various ratios detected using 2,7-dichlorofluorescin diacetate (10 μM) as the fluorescent probe. (i) Schematic diagram of the electronic structure of monomeric and aggregated PcN4-BA and their possible photophysically activated process.

Zinc(ii) phthalocyanines are second-generation PSs for PDT. Compared to porphyrins, zinc(ii) phthalocyanines usually have stronger light absorption in the far-red/near-infrared (NIR) region and higher ROS generation efficiencies.^12^–^17^ Introduction of quaternary ammonium groups on phthalocyanines is an attractive approach to improve hydrophilicity and the affinity for the outer membrane of bacteria.^18^,^19^ In addition, recent studies have suggested that boronic acid groups are very useful for bacterial targeting because of their ability to form a pair of covalent bonds with glycans on the surface of bacteria.^20^–^22^ Therefore, the 3-{*N*-(4-boronobenzyl)-*N*,*N*-dimethylammonium}phenoxy-substituted zinc(ii) phthalocyanine PcN4-BA (Fig. 1a) was herein designed to be a new PS for antimicrobial PDT. The synthesis of PcN4-BA is demonstrated in the ESI.†

## Results and discussion

First, we measured the steady-state absorption and emission spectra of PcN4-BA in mixtures of various DMF : H_2_O ratios (Fig. 1b and c). Compared to the sharp and intense absorption features of PcN4-BA in DMF, the absorption spectra were significantly changed with increasing H_2_O fraction. In H_2_O, the spectra extend to over 800 nm with a distinct decrease in the extinction coefficient and relatively broad bands. Along with this absorption spectral change, the fluorescence emission gradually attenuated. These distinct absorption and emission spectral changes indicate that PcN4-BA undergoes a significant electronic structural change driven by their aggregation under the polar condition,^23^,^24^ which was clearly observed by transmission electron microscopy (*vide infra*). Here, the broadening of absorption bands with NIR tailing over 800 nm well manifests an increased density of electronic states by intense intermolecular interaction in the PcN4-BA aggregates, where additional collective vibrations promote non-radiative decay processes and result in the lack of fluorescence in H_2_O.^23^

Next, to understand a change of excited state dynamics upon the molecular aggregation, we measured nanosecond transient absorption (ns-TA) spectra of PcN4-BA (Fig. 1d and e). Although the same concentration of PcN4-BA was contained in DMF and H_2_O solvents, the ns-TA spectra showed significantly different features. Compared to the TA spectra of PcN4-BA in DMF, the corresponding spectra in H_2_O showed distinct broad negative bleaching with attenuation of signal by a factor of ten, reflecting that the molecular aggregation causes intense intermolecular interaction and a large change in the electronic structure.

Furthermore, with increasing H_2_O fraction, the decay of ns-TA signals accelerated (Fig. 1f and g), indicating a decrease in the triplet-state lifetime. This result indicates that a reduced energy gap and enhancement of intersystem crossing (ISC) between the lowest triplet excited and singlet ground states by the intense intermolecular interaction in the aggregates stimulate their non-radiative decay processes. Here, it is noteworthy that the signal for ROS generation is intensified with increasing H_2_O fraction (Fig. 1h), which directly describes that more triplet excitons are being produced in the aggregated PcN4-BA than in the monomer by photoexcitation. This is well matched with the ns-TA data manifesting the enhanced ISC by the aggregation. The enhanced ROS generation in PcN4-BA aggregates was also observed in aqueous solutions containing different concentrations of Cremophor EL (Fig. S1†). This enhanced ROS generation can be understood by the aggregation-induced enhanced ISC process (Fig. 1i).^24^ The large intermolecular interaction in the aggregates increases the density of electronic states which significantly reduces the energy gap between the excited singlet and triplet states. Thus, the enhanced energy overlap between excited singlet and triplet states caused by the aggregation promotes ISC, and the subsequent increase in triplet excitons results in the improvement in ROS generation. Although the lifetimes of triplet excited states are shortened by aggregation, the TA signal is prolonged by more than 200 μs in H_2_O. This time scale is sufficient for aggregates to collide with other species, such as oxygen, by Brownian motion (ESI†).^25^

**Fig. 2:**
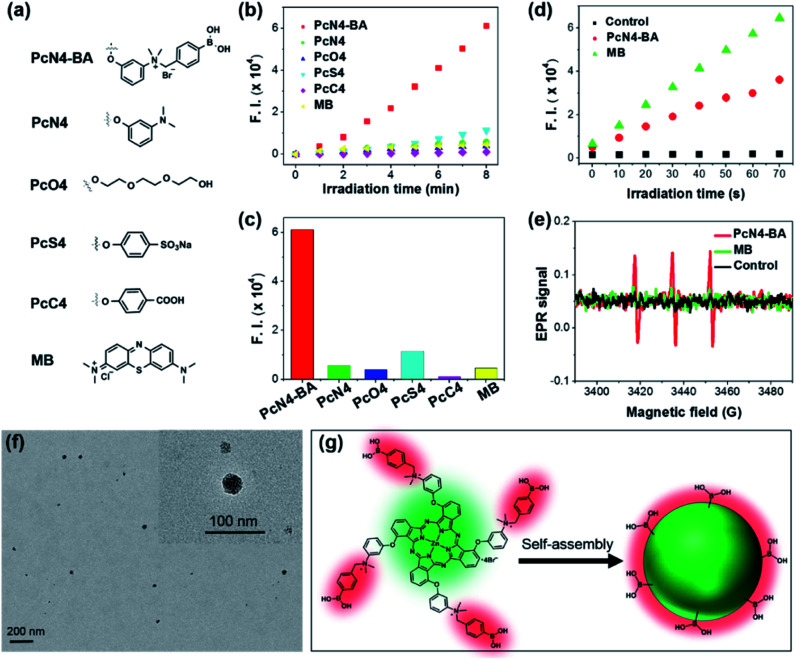
Highly efficient ROS generation and nanostructure self-assembly by PcN4-BA in water. (a) Molecular structures of different PSs. For the phthalocyanine derivatives, only their substituted groups (R, same position as PcN4-BA as shown in Fig. 1a) are displayed. (b) ROS generation plot of different PSs (all at 10 μM) in water detected using 2,7-dichlorofluorescin diacetate (10 μM) as the fluorescent probe. (c) Fluorescence intensity of the ROS probe after incubation with different PSs and irradiation with light for 8 min. (d) Superoxide anion generation of PcN4-BA and MB in water. (e) Singlet oxygen generation of PcN4-BA and MB in water determined by electron paramagnetic resonance (EPR) analysis. The light irradiation time was 10 min. (f) The morphology of PcN4-BA nanoparticles determined by transmission electron microscopy. (g) Schematic illustration of the nanostructured self-assembly of PcN4-BA.

Compared to four other phthalocyanine derivatives (PcN4, PcO4, PcS4, and PcC4) with different substituent groups (Fig. 2a), PcN4-BA promoted ROS generation much more efficiently (Fig. 2b and c). In addition, the ROS generation induced by PcN4-BA was approximately 13 times higher than that induced by the clinically used PS methylene blue (MB). The detection of the superoxide anion (Fig. 2d) and singlet oxygen (Fig. 2e) indicated that PcN4-BA is involved in both type I and type II photochemical reactions.^26^,^27^

More interestingly, PcN4-BA self-assembled uniform nanostructures in water. From dynamic light scattering tests (Fig. S2†), we found that PcN4-BA in water could form dispersions of stable nanoparticles with average sizes ranging from 40 to 100 nm. In Fig. 2f, transmission electron microscopy images show that nanostructured PcN4-BA assemblies are nearly spherical. We speculate that intermolecular π–π interactions induced by the phthalocyanine macrocyclic structure promotes stacking of the PcN4-BA molecules, and surface hydrophilic interactions (*e.g.*, electrostatic repulsion) between the 3-{*N*-(4-boronobenzyl)-*N*,*N*-dimethylammonium}phenoxy moieties and water sufficiently stabilizes the individual nanoparticles (Fig. 2g).

**Fig. 3:**
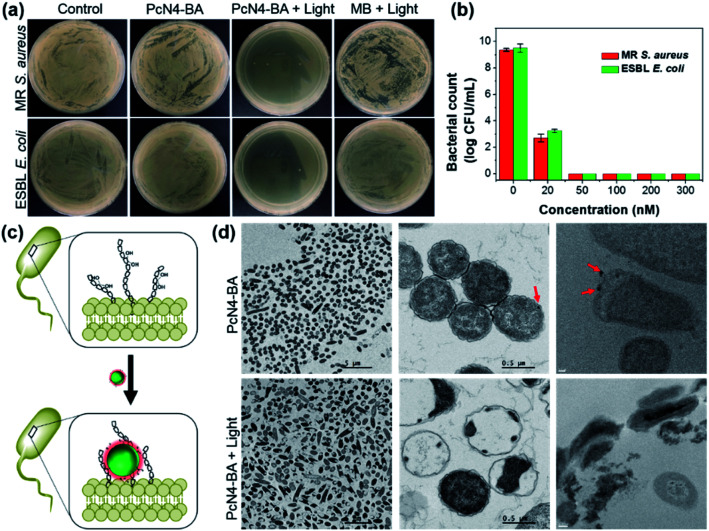
Photodynamic antibacterial effect of PcN4-BA. (a) Plate photographs for MR *S. aureus* and ESBL *E. coli* with the indicated treatments. PcN4-BA and MB were at the same concentration (50 nM). (b) MR *S. aureus* and ESBL *E. coli* photoinactivation in the presence of different concentrations of PcN4-BA and laser irradiation (655 nm, 0.4 W cm^–2^, 10 min). (c) Schematic illustration of the binding of PcN4-BA nanoparticles to the surface of bacteria through the formation of covalent bonds between boronic acids and glycans. (d) TEM images (left and middle column) and cryo-TEM images (right column) of *E. coli* cells with the indicated treatments. Red arrows indicate PcN4-BA nanoparticles. Scale bars for cryo-TEM images are 0.1 μm (top) and 0.2 μm (bottom).

Encouraged by the above uncommon findings, we subsequently evaluated the photodynamic antibacterial efficacy of PcN4-BA. In this investigation, two representative common bacterial strains, *Staphylococcus aureus* and *Escherichia coli*, and two representative antibiotic-resistant bacterial strains, methicillin-resistant *S. aureus* (MR *S. aureus*) and extended spectrum beta-lactamase *E. coli* (ESBL *E. coli*), were selected as model targets. As shown in Fig. 3a, b and S3,† PcN4-BA induced a significant decrease in bacterial colonies upon light irradiation. For example, at a concentration of 50 nM and with 655 nm laser irradiation (0.4 W cm^–2^) for 10 min, PcN4-BA inhibited the growth of MR *S. aureus* and ESBL *E. coli* by almost 100%. However, under the same conditions as PcN4-BA, MB did not show significant photodynamic antibacterial efficiency. We also found that PcN4-BA showed much higher antibacterial efficacy than those of protoporphyrin IX (PPIX) and 4,4′,4′′,4′′′-(porphine-5,10,15,20-tetrayl)tetrakis(benzenesulfonic acid) (TPPS) upon light irradiation (Fig. S4†). More importantly, compared to PcN4-M, which has only quaternary ammonium groups (Fig. S5a†), PcN4-BA showed a much better phototherapeutic index with higher bacterial inactivation under light irradiation (Fig. S5b†) but lower bacterial inactivation in the dark (Fig. S5c†). We speculated that the boronic acid groups of PcN4-BA nanoparticles probably promote the binding of PcN4-BA to the surface of bacteria (Fig. 3c), and then the highly efficient ROS generation induced by light irradiation likely contributes to its high antibacterial effect. Both TEM images and cryo-TEM images confirmed that PcN4-BA nanoparticles bind to the surface of bacteria and that the cell membranes are disrupted after treatment with both PcN4-BA and light irradiation (Fig. 3d).

The cytotoxicity of PcN4-BA against normal mammalian cells was also evaluated for further potential applications. In this study, a human diploid lung fibroblast cell line (WI-38 VA-13 subline 2RA) was employed as the representative cell. Fig. S6† shows that PcN4-BA had no significant cytotoxicity even at a high concentration (5000 nM) that was 100-fold higher than the concentration used for bacterial inactivation. These results suggest that the introduction of boronic acids is an important strategy to enable PcN4-BA to function as an efficient and safe PS for antimicrobial PDT applications.

## Conclusions

In conclusion, we have developed an uncommon PS, PcN4-BA, that exhibits an aggregation-enhanced photodynamic effect. In water, this novel PS forms uniform sphere-like nanoparticles *via* a self-assembling process and displays highly efficient ROS generation through both type I and type II photochemical mechanisms. In addition, PcN4-BA displays excellent photodynamic antimicrobial activity against both common and antibiotic-resistant bacterial strains.

## Conflicts of interest

There are no conflicts to declare.

## Supplementary Material

Supplementary information
