# Supplementary material for: A boronic acid-functionalized phthalocyanine with an aggregation-enhanced photodynamic effect for combating antibiotic-resistant bacteria
Source: Chem Sci. 2020 May 18;11(22):5735–9. doi: 10.1039/d0sc01351j (PMC7425080; doi:10.1039/d0sc01351j)
Supplement: Supplementary file 1 [file SC-011-D0SC01351J-s001.pdf]

Electronic Supplementary Material (ESI) for Chemical Science.
This journal is © The Royal Society of Chemistry 2020

Supporting Information

A boronic acid-functionalized phthalocyanine with an aggregation-enhanced photodynamic effect for combating antibiotic-resistant bacteria

Eunhye Lee,[‡b] Xingshu Li,[*‡a] Juwon Oh,[‡c] Nahyun Kwon,[b] Gyoungmi Kim,[b] Dongho Kim[*c] and Juyoung Yoon[*b]

[a] College of Chemistry, State Key Laboratory of Photocatalysis on Energy and Environment, Fujian Provincial Key Laboratory of Cancer Metastasis Chemoprevention and Chemotherapy, Fuzhou University, Fuzhou 350108, China.

[b] Department of Chemistry and Nanoscience,Ewha Womans University, Seoul 120-750, Republic of Korea.

[c] Spectroscopy Laboratory for Functional π-Electronic Systems and Department of Chemistry, Yonsei University, Seoul 120-749, Republic of Korea.

*Corresponding authors, e-mail address:
xingshuli@fzu.edu.cn; dongho@yonsei.ac.kr; jyoon@ewha.ac.kr

‡These authors contributed equally to this work.

Materials and instruments

N,*N*-dimethyl formamide (DMF), n-pentanol, dimethyl sulfoxide (DMSO), potassium carbonate, 1,8-diazabicyclo-[5.4.0]undec-7-ene (DBU), zinc acetate, 3-nitrophthalonitrile, 3-(dimethylamino)phenol, 4-(bromomethyl)phenylboronic acid, 2,7-dichlorofluorescin diacetate (DCFH-DA), methylene blue (MB), 2,2,6,6-tetramethylpiperidine (TEMP), Cremophor EL (CEL), hydroethidine (DHE), Protoporphyrin IX (PPIX) and 4,4',4'',4'''-(Porphine-5,10,15,20-tetrayl)tetrakis(benzenesulfonic acid) (TPPS), ctDNA and 3-(4,5-dimethyl-2-thiazolyl)-2,5-diphenyl-2H-tetrazolium bromide (MTT) were purchased from Sigma-Aldrich Korea. Four control phthalocyanines (PcO4[1], PcS4[2], PcC4[3] and PcN4-M[4]) were prepared according to our previously described procedures.

Absorption spectra were recorded on an Evolution 201 UV/VIS Spectrometer (Thermo Fisher Scientific). Fluorescence emission spectra were obtained on an FS-2 spectrophotometer (Scinco). ^{1}H NMR spectra were obtained by a Bruker AM 300 MHz. High-resolution mass spectra (HRMS) were determined using a fast atom bombardment with electrospray ionization (FAB, EI) JMS 700 (JEOL; Daegu Center of the Korea Basic Science Institute (KBSI)) or an Exactive Plus Orbitrap (Thermo Fisher Scientific). Dynamic light scattering (DLS) was measured using a Nano-ZS instrument (Malvern). Electron paramagnetic resonance (EPR) spectra were obtained on an EMX-plus (Bruker; Western Seoul Center of KBSI). Nanosecond TA spectra were obtained using an optical parametric oscillator system (Continuum, Surelite OPO) pumped by a Q-switched Nd:YAG laser (Continuum, Surelite II-10), and signals were recorded by a 500 MHz digital storage oscilloscope (Lecroy, WaveRunner 6050A). Transmission electron microscopy (TEM) images were obtained using a JEM-2100F (JEOL) or a JEM1011 (JEOL). Cryo-TEM images were recorded using a CryoTecnai F20 G2 (FEI; Korea Institute of Science and Technology, 200 kV).

Synthesis and characterization of PcN4

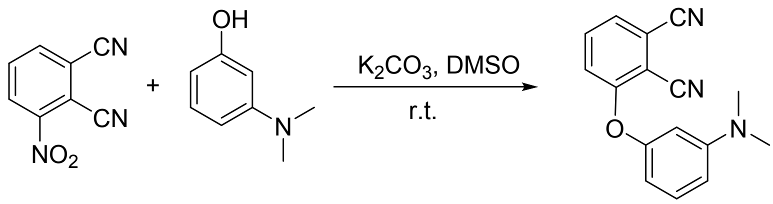

A mixture of 3-nitrophthalonitrile (2.61 g, 15 mmol), 3-(dimethylamino)phenol (2.06 g, 15 mmol), and anhydrous K_2CO_3 (4.14 g, 30 mmol) in DMSO (solvent, 30 mL) was stirred at room temperature for 72 h under an atmosphere of nitrogen. The reaction mixture was poured into water (150 mL) and allowed to stand for 3 h. The resulting brown solid was filtered and washed with water and then dried in vacuo. The product was afforded as a light brown solid (2.56 g, 65% yield). ^{1}H NMR (300 MHz, DMSO-d_6) δ 7.84 – 7.74 (m, 2H), 7.29 – 7.20 (m, 2H), 6.67 – 6.39 (m, 3H), 2.90 (s, 6H). HRMS (EI): m/z Calcd for $C_{16}H_{13}N_3O$, $[M]^+$ 263.1059, found 263.1059.

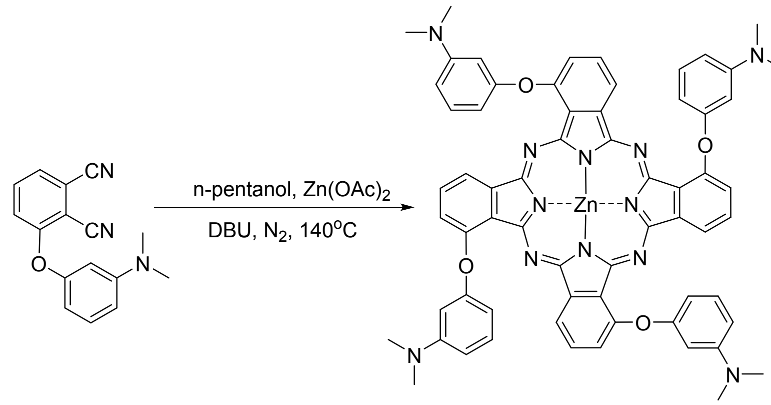

Next, 3-[3-(dimethylamino)phenoxy]phthalonitrile (1 g, 3.8 mmol) in n-pentanol (40 mL) was stirred at 90 °C under an atmosphere of nitrogen for 30 min, and then zinc acetate (0.7 g, 3.8 mmol) and 1,8-diazabicyclo-[5.4.0]undec-7-ene (DBU) (2 mL, 13.4 mmol) were added. The resulting mixture was stirred at 140 °C for 48 h (reflux). After being cooled, the reaction mixture was poured into water (200 mL) and allowed to stand for 3 h. The resulting green solid was filtered and washed with water and methanol and then dried in vacuo. Then, the crude product was first purified by silica gel column chromatography using CH_2Cl_2 and ethyl acetate as the eluent to give a green mixture. The mixture was concentrated under reduced pressure and purified by size exclusion chromatography on a Bio-Beads S-X3 column using DMF as the eluent. The crude product was further purified by recrystallization from a mixture of $CHCl_3$ and hexane to afford a green solid (1.275 g, 30% yield). ^{1}H NMR (300 MHz, DMSO-d_6) δ 9.31 – 8.72 (m, 4H), 8.27 – 8.09 (m, 4H), 7.98 – 7.75 (m, 4H), 7.43 – 7.03 (m, 8H), 6.60 – 6.50 (m, 8H), 3.03 – 2.80 (m, 24H). HRMS (FAB): m/z Calcd for $C_{64}H_{52}N_{12}O_4Zn$, $[M+H]^+$ 1117.3599, found 1117.3600.

Synthesis and characterization of PcN4-BA

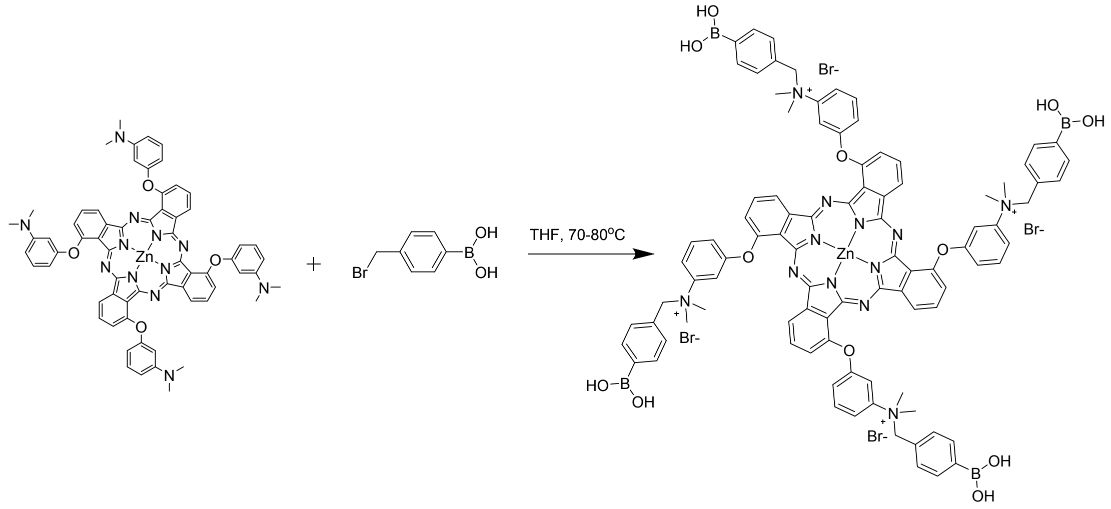

PcN4 (200 mg, 0.179 mmol) and 4-(bromomethyl)phenylboronic acid (306 mg, 1.432 mmol) in THF (solvent, anhydrous, 50 mL) were stirred at temperature (70-80 °C) under an atmosphere of nitrogen for 48 h (reflux). After the reaction, precipitation occurred in a round bottom flask, and green solvent (containing PcN4, PcN4-BA) was removed by a glass pipet. After the sample was cooled, THF (100-150 mL) was poured into the resulting mixture (precipitate), which was then filtered with THF (membrane filtration, 2 times). Then, filtered green solid (still containing PcN4 and PcN4-BA) was dissolved in MeOH (4-5 mL, sonication), and $CHCl_3$ was added (200-300 mL). The mixture stood for a few hours, and a green solid was recrystallized. The green solid was filtered and washed with $CHCl_3$ and then dried in vacuo (110 mg, 31% yield). ^{1}H NMR (300 MHz, DMSO-d_6) δ 9.37 – 8.64 (m, 4H), 8.35 - 8.31 (m, 4H), 8.13 - 8.02 (m, 4H), 8.00 – 7.83 (m, 8H), 7.73 – 7.54 (m, 16H), 7.38 – 7.30 (m, 4H), 7.12 - 7.07 (m, 4H) 5.13 – 4.93 (m, 8H), 3.74 – 3.57 (m, 24H). HRMS (ESI): m/z Calcd for $C_{92}H_{84}B_4N_{12}O_{12}Zn[M-4Br]^{4+}$ 414.4002, found 414.4007.

ns-transient absorption (TA) spectra

Nanosecond TA spectra were obtained using nanosecond flash photolysis. Specifically, a tunable excitation pulse was generated using an optical parametric oscillator system (Continuum, Surelite OPO) that was pumped with 355 nm light from the third-harmonic output of a Q-switched Nd:YAG laser (Continuum, Surelite II-10). The duration of the excitation pulse was ca. 6 ns, and the pulse energy was ca. 2 mJ/pulse. A CW Xe lamp (150 W) was used as the probe light source for the TA measurement. The probe light was collimated at the sample cell and was spectrally resolved using a 15 cm monochromator (Acton Research, SP150) equipped with a 600 grooves/mm grating after the light passed the sample. The spectral resolution was approximately 3 nm for the TA experiment. The light signal was detected using an avalanche photodiode (APD). The output signal from the APD was recorded using a 500 MHz digital storage oscilloscope (Lecroy, WaveRunner 6050A) for temporal profile measurement. Since the triplet-state dynamics of molecules in solution are strongly dependent on the dissolved oxygen concentration, we attempted to remove oxygen by bubbling with Ar gas for 30 min.

Calculated collisions of aggregates in H_2O over 100 μs

Fraction of oxygen molecules in H_2O under saturated conditions at RT = 4.68 x 10^{-6}
Collisions of particles with small surrounding molecules, such as H_2O and O_2 = 10^{20} s^{-1}
Considering 100 μs = 10^{-4} s
▸ Collisions of particles in H_2O during 100 μs: (4.68 x 10^{-6}) x (10^{-4})s x (10^{20})s^{-1} = 4.68 x 10^{10}

ROS detection

DCFH-DA was used as a probe for reactive oxygen species (ROS). In the presence of ROS, DCFH-DA forms dichlorofluorescein (DCF), which emits bright fluorescence at approximately 522 nm. Briefly, photosensitizers (PSs, 10 μM) and DCFH-DA (10 μM) were dissolved at different water:DMF ratios. Similarly, PSs (10 μM) and DCFH-DA (10 μM) were dissolved in water with different concentrations of CEL (0%-1%). Measurements were performed by irradiating mixtures with 500 W halogen light. A water tank was used for cooling. Fluorescence spectra were then recorded using an FS-2 spectrophotometer (Scinco). Samples were excited at 504 nm and measured at 510-900 nm, and the emission intensity at 522 nm was used.

Superoxide anion detection

DHE was used as a probe for the measurement of superoxide anion. DHE can be oxidized by superoxide to form ethidium, which can emit red fluorescence at approximately 570 nm upon DNA intercalation.

To prepare an aqueous DNA solution (250 μg/mL), 5 mg of DNA sodium salt from calf thymus (ctDNA) was dissolved in water for 12 h. Then, PSs (PcN4-BA and MB, 10 μM) and DHE (50 μM) were dissolved in the DNA solution. A DNA solution containing only hydroethidium was used as a control. Measurements were performed by irradiating mixtures with 500 W halogen light. A water tank is used for cooling. After a delay, fluorescence spectra were recorded using an FS-2 spectrophotometer (Scinco). Samples were excited at 510 nm and measured at 520-900 nm, and the emission intensity at 570 nm was used.

Singlet oxygen detection

singlet oxygen measurement was carried out by EPR analysis using a spin-trapping method at room temperature. TEMP was used as a spin probe to indicate the generation of singlet oxygen, resulting in the formation of (2,2,6,6-tetramethylpiperidin-1-yl)oxyl (TEMPO). Samples were prepared by mixing 100 μL of PSs (PcN4-BA and MB, 0.2 mM) in water and 20 μL of TEMP (300 mM) in water. A water solution containing only TEMP was used as the control. EPR signals were recorded by adding samples through a capillary tube under 655 nm light irradiation at 0.4 W/cm^2 (0-10 min).

Photodynamic antibacterial effect

E. coli O157:H7 (Gram negative, ATCC 43894), *S. aureus* (Gram positive, ATCC 25923), ESBL *E. coli* (Gram negative, ATCC BAA-198), and MR *S. aureus* (Gram positive, CCRAM 3696) were used for antibacterial testing.

Each strain of bacteria was grown for 12 h on a plate by streaking to obtain a single colony. An isolated colony from an agar plate of each bacterial strain was grown for a few hours in

4 mL of broth culture (Luria-Bertani, LB Broth) at 37 °C until the optical density (OD_{600}) reached 1. Then, the culture solution was centrifuged at 5000 rpm. The culture supernatant was removed, and the precipitate was washed with PBS twice. One milliliter of fresh PBS was added to precipitate to make a bacterial stock solution.

Different concentrations (0-300 nM) of PSs were used to treat bacterial solutions at a concentration of 8.0 x 10^7 CFU/mL for *E. coli* and 1.0 x 10^8 CFU/mL for *S. aureus* (diluted 10 times from stock solution). Then, the mixtures of PSs and bacterial solution were incubated for 2 h with rotation at 37 °C. After incubation, 200 μL of the mixture was irradiated with a 655 nm laser (0.4 W/cm^2) for 10 min. The irradiated mixture was diluted 10 times with PBS, and 200 μL of the mixture was added onto an agar plate spreading over the surface. Then, the plates were incubated overnight at 37 °C. The number of living bacteria was determined by the Miles and Misra plate method.

$$\text{Number of Bacteria(CFU/mL)} = \frac{\text{The number of colonies on a plate}}{\text{Volume (mL)}} \times \text{Dilution factor}$$

MTT test

WI-38 VA-13 subline 2RA cells were seeded in 96-well plates with MEM culture medium. After 24 h, the cells were incubated with samples (0 nM, 50 nM, 100 nM, 200 nM, 300 nM, 500 nM, 1000 nM, or 5000 nM) for 2 h and then washed with buffer. After adding fresh MEM culture media, the cells were incubated for another 24 h. The cell viability was evaluated with the MTT method using a Spectramax Microwell plate reader.

TEM detection

E. coli cells were grown for 12 h on a plate by streaking to obtain a single colony. An isolated colony from an agar plate of each bacterial strain was grown for a few hours in 4 mL of LB broth at 37 °C until the OD_{600} reached 1. Then, the culture solution was centrifuged three times for 3 min each at 5000 rpm. The culture supernatant was removed, and the precipitate was washed with PBS three times. Each bacterial solution was incubated with PcN4-BA (500 nM) at 37 °C for 2 h. For irradiated samples, 1000 μL of the mixture was irradiated with a laser (655 nm, 0.4 W/cm^2) for 10 min. Then, the mixture was centrifuged, and the supernatant was discarded.

Samples were fixed for 12 h in 2% glutaraldehyde-paraformaldehyde in 0.1 M phosphate buffer (pH 7.4) and washed in 0.1 M phosphate buffer. They were postfixed with 1% OsO_4 dissolved in 0.1 M PBS for 2 h, dehydrated in an ascending gradual series (50-100%) of ethanol and infiltrated with propylene oxide. Specimens were embedded by a Poly/Bed 812 kit (Polysciences). After pure fresh resin embedding and polymerization in a 65 °C electron microscope oven (TD-700, Dosaka, Japan) for 24 h, approximately 200~250 nm-thick sections were initially cut and stained with toluidine blue (Sigma, T3260) for light microscopy. A 70 nm thin section was double stained with 6% uranyl acetate (EMS, 22400, 20 min) and lead citrate (Fisher, 10 min) for contrast staining. These sections were cut by a Leica EM UC-7 (Leica Microsystems, Austria) with a diamond knife (Diatome) and transferred to copper and nickel grids. All thin sections were observed by TEM (JEM-1011, JEOL, Japan) at an acceleration voltage of 80 kV.

Cryo-TEM detection

E. coli cells were grown for 12 h on a plate by streaking to obtain a single colony. An isolated colony from an agar plate of each bacterial strain was grown for a few hours in 4 mL of LB broth at 37 °C until the OD_{600} reached 1. Then, the culture solution was centrifuged three times for 3 min each at 5000 rpm. The culture supernatant was removed, and the precipitate was washed with PBS three times. Each bacterial solution was incubated with PcN4-BA (500 nM) at 37 °C for 2 h. For irradiated samples, 1000 μL of the mixture was irradiated with a laser (655 nm, 0.4 W/cm^2) for 10 min. Then, the mixture was centrifuged, and the supernatant was discarded.

For postfixation, 2% paraformaldehyde was added to precipitated cells, which were fixed overnight at 4 °C. Then, bacterial cells were suspended in 2% aqueous OsO_4 (0.1 M PBS, pH 7.4), washed 3 times for 5 min in 0.1 M PBS, and dehydrated 2 times each at different concentrations of ethanol (50%, 70%, 95%, 100%) for 15 min and in propylene oxide 2 times for 15 min. The samples were put into propylene oxide/Epon resin overnight and kept in fresh 100% Epon resin for 1-2 h for penetration. The samples were embedded and polymerized in BEEM capsules at 60 °C for over 12 h. Then, thick sections (1 micron) were scanned to find the area of sample, and ultrathin sections (~70 nm) were then obtained by a Leica Ultracut (Leica company) to be placed on copper grids. The samples in the copper grids were electrostained with uranyl acetate for 5 min and with lead citrate for 2 min. The samples were detected using a cryo-TEM (FEI;CryoTecnai F20, 200 kV).

Supplementary figures

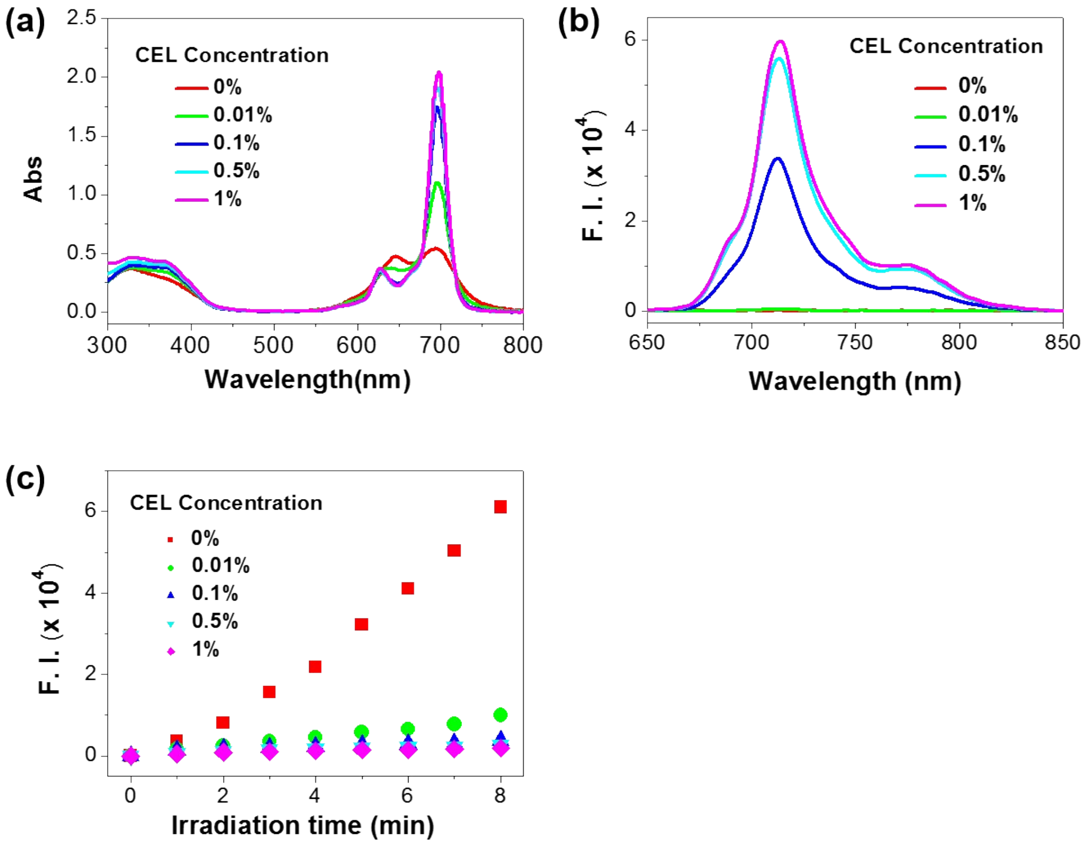

Figure S1. (a) Absorption and (b) emission (excited at 635 nm) spectra of PcN4-BA (10 μM) in water with different concentrations of CEL. (c) The ROS generation plot of PcN4-BA in water with different concentrations of CEL. CEL is a common surfactant that is often utilized for improving the water solubility and disaggregation of photosensitizers.[5-8]

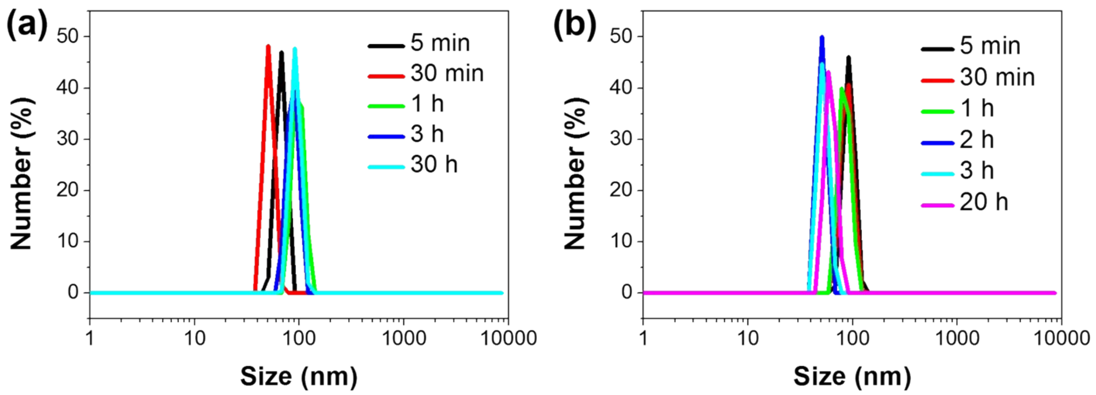

Figure S2. Size distribution of PcN4-BA at concentration of (a) 10 μM and (b) 20 μM in water after aging for different time detected by DLS.

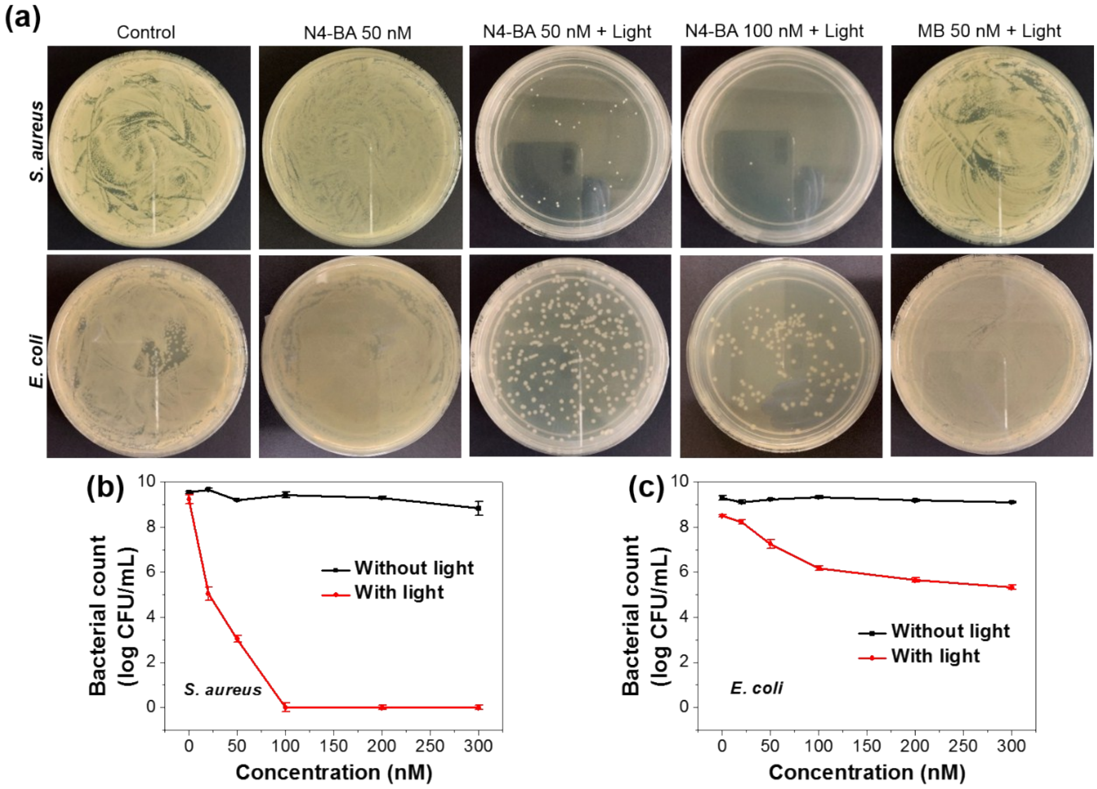

Figure S3. (a) Plate photographs for *S. aureus* and *E. coli* on LB agar plate treated with PcN4-BA (50 nM) in dark, PcN4-BA (50 or 100 nM) under light irradiation, and MB (50 nM) under light irradiation. Control groups are bacteria with only light treatment. Light condition: 655 nm laser, 0.4 W/cm^2, 10 min. (b) *S. aureus* and (c) *E. coli* inactivation in the presence of different concentrations of PcN4-BA with and without light irradiation (655 nm, 0.4 W/cm^2, 10 min).

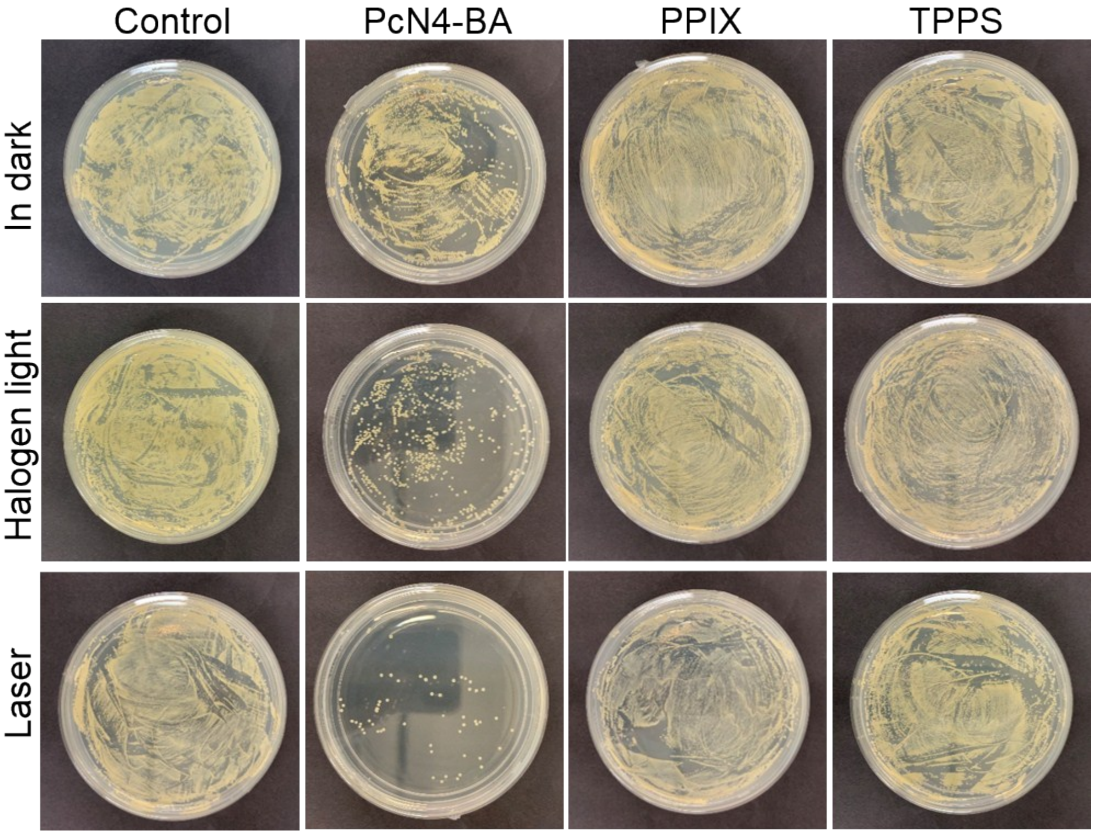

Figure S4. Plate photographs for *S. aureus* on LB agar plate treated with PcN4-BA, PPIX, and TPPS (all at 50 nM) in dark or under light irradiation. Control groups are bacteria without treatment. Halogen light condition: 0.2 W/cm^2, 10 min. Laser light condition: 655 nm, 0.4 W/cm^2, 10 min.

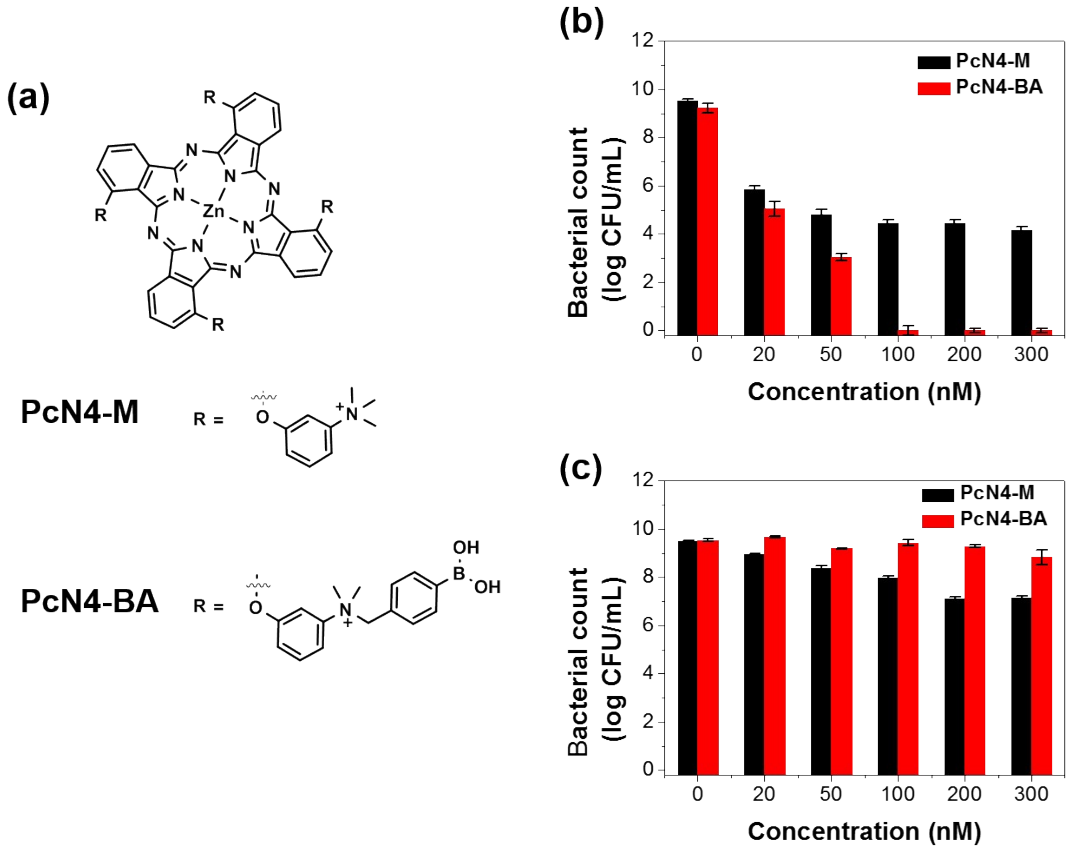

Figure S5. (a) Chemical structure of PcN4-M and PcN4-BA. Here, only one of the possible C_{4h} isomers is displayed for the tetra-substituted phthalocyanine. (b) *S. aureus* photoinactivation in the presence of different concentrations of PcN4-M and PcN4-BA and light irradiation (655 nm, 0.4 W/cm^2, 10 min). (c) *S. aureus* inactivation in the presence of different concentrations of PcN4-M and PcN4-BA in dark.

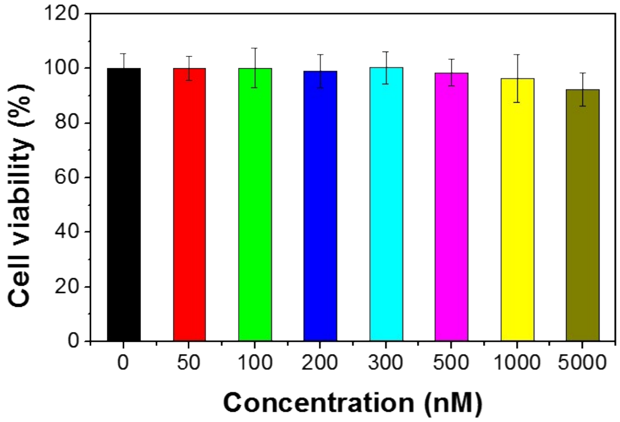

Figure S6. Cytotoxic effects of PcN4-BA on WI-38 VA-13 subline 2RA cells.

References

(1) Li, X.; Kim, C. -y.; Lee, S.; Lee, D.; Chung, H. -M.; Kim, G.; Heo, S. -H.; Kim, C.; Hong, K. -S.; Yoon, J. Nanostructured phthalocyanine assemblies with protein-driven switchable photoactivities for biophotonic imaging and therapy. *J. Am. Chem. Soc.* **2017**, *139*, 10880-10886.

(2) Li, X. -S.; Ke, M. -R.; Huang, W.; Ye, C. -H.; Huang, J. -D. A pH-Responsive Layered Double Hydroxide (LDH)-Phthalocyanine Nanohybrid for Efficient Photodynamic Therapy. *Chem. -Eur. J.* **2015**, *21*, 3310-3317.

(3) Li, X.; Fan, H.; Guo, T.; Bai, H.; Kwon, N.; Kim, K. H.; Yu, S.; Cho, Y.; Kim, H.; Nam, K. T.; Yoon, J.; Zhang, X. -B.; Tan, W. Sequential protein-responsive nanophotosensitizer complex for enhancing tumor-specific therapy. *ACS Nano* **2019**, *13*, 6702-6710.

(4) Li, X.; Jeong, K.; Lee, Y.; Guo, T.; Lee, D.; Park, J.; Kwon, N.; Na, J. –H.; Hong, S. K.; Cha, S. –S.; Huang, J. –D.; Choi, S.; Kim, S.; Yoon, J. Water-soluble phthalocyanines selectively bind to albumin dimers: a green appoach toward enhancing tumor-targeted photodynamic therapy. *Theranostics* **2019**, *9*, 6412-6423.

(5) Li, X.; Peng, X. -H.; Zheng, B. -D.; Tang, J.; Zhao, Y.; Zheng, B. -Y.; Ke, M. -R.; Huang, J. -D. New application of phthalocyanine molecules: from photodynamic therapy to photothermal therapy by means of structural regulation rather than formation of aggregates. *Chem. Sci.* **2018**, *9*, 2098-2104.

(6) Zhang, Y.; Lovell, J. F. Recent applications of phthalocyanines and naphthalocyanines for imaging and therapy. *Wiley Interdiscip. Rev.: Nanomed. Nanobiotechnol.* **2017**, *9*, e1420.

(7) Lau, J. T.; Lo, P. C.; Jiang, X. J.; Wang Q.; Ng, D. K. A dual activatable photosensitizer toward targeted photodynamic therapy. *J. Med. Chem.* **2014**, 57, 4088–4097.

(8) Ji, D.; Wang, X. -Z.;Yang, L. -F.; Li, S. -C.; Hu, Q. -Y.; Li, X. Zheng, B. -Y.; Ke, M. -R.; Huang, J. -D. Size-tunable targeting-triggered nanophotosensitizers based on self-assembly of a phthalocyanine-biotin conjugate for photodynamic therapy. *ACS Appl. Mater. Interfaces* **2019**, *11*, 36435-36443.
